# Arteriovenous Malformations Treated With Frameless Robotic Radiosurgery Using Non-Invasive Angiography: Long-Term Outcomes of a Single Center Pilot Study

**DOI:** 10.3389/fonc.2020.570782

**Published:** 2020-11-30

**Authors:** Ryan Kelly, Anthony Conte, M. Nathan Nair, Jean-Marc Voyadzis, Amjad Anaizi, Sean Collins, Christopher Kalhorn, Andrew Stemer, Jeffery Mai, Rocco Armonda, Jonathan Lischalk, Frank Berkowitz, Vikram Nayar, Kevin McGrail, Brian Timothy Collins

**Affiliations:** ^1^ Georgetown University School of Medicine, Washington, DC, United States; ^2^ Department of Neurosurgery, Medstar Georgetown University Hospital, Washington, DC, United States; ^3^ Department of Radiation Medicine, Medstar Georgetown University Hospital, Washington, DC, United States; ^4^ Department of Neurology, Medstar Georgetown University Hospital, Washington, DC, United States; ^5^ Department of Radiology, Medstar Georgetown University Hospital, Washington, DC, United States; ^6^ Department of Neurosurgery, MedStar Washington Hospital Center, Washington, DC, United States

**Keywords:** arteriovenous malformations, CyberKnife, radiosurgery, neuroradiology, pilot study

## Abstract

**Objective:**

CT-guided, frameless robotic radiosurgery is a novel radiotherapy technique for the treatment of intracranial arteriovenous malformations (AVMs) that serves as an alternative to traditional catheter-angiography targeted, frame-based methods.

**Methods:**

Patients diagnosed with AVMs who completed single fraction frameless robotic radiosurgery at Medstar Georgetown University Hospital between July 20, 2006 – March 11, 2013 were included in the present study. All patients received pre-treatment planning with CT angiogram (CTA) and MRI, and were treated using the CyberKnife radiosurgery platform. Patients were followed for at least four years or until radiographic obliteration of the AVM was observed.

**Results:**

Twenty patients were included in the present study. The majority of patients were diagnosed with Spetzler Martin Grade II (35%) or III (35%) AVMs. The AVM median nidus diameter and nidal volume was 1.8 cm and 4.38 cc, respectively. Median stereotactic radiosurgery dose was 1,800 cGy. After a median follow-up of 42 months, the majority of patients (81.3%) had complete obliteration of their AVM. All patients who were treated to a total dose of 1800 cGy demonstrated complete obliteration. One patient treated at a dose of 2,200 cGy developed temporary treatment-related toxicity, and one patient developed post-treatment hemorrhage.

**Conclusions:**

Frameless robotic radiosurgery with non-invasive CTA and MRI radiography appears to be a safe and effective radiation modality and serves as a novel alternative to traditional invasive catheter-angiography, frame-based methods for the treatment of intracranial AVMs. Adequate obliteration can be achieved utilizing 1,800 cGy in a single fraction, and minimizes treatment-related side effects.

## Background

Intracranial arteriovenous malformations (AVM) are rare, congenital lesions consisting of a series of direct, high-flow connections from arteries to veins without the presence of intervening capillaries. This high-flow shunting of blood may lead to venous dilation, engorgement, and ultimately vessel rupture. Rupture of these lesions is one of the leading causes of intracranial hemorrhage in the adolescent and young adult population, and results in high rates of neurologic morbidity or mortality. To prevent hemorrhage, a treatment must safely obliterate the AVM without leaving a residual. Arteriovenous malformations are highly variable. Multidisciplinary evaluation helps with appropriate patient selection. The various options of management should be considered for each patient, to determine which option is safest: microsurgical resection with or without preoperative embolization, radiosurgery, or observation. Treatment of high-grade AVMs carries a significant risk of neurologic complications, and nonintervention may be favored in these cases (1).

Stereotactic radiosurgery (SRS) has advanced and become a more ubiquitous form of treatment over the last several decades as an effective treatment for both ruptured and unruptured AVMs given its high rate of nidus obliteration, minimal invasiveness, and low side effect profile. Obliteration rates between 50% and 80% over 2 to 5 years have been observed with SRS treatment of AVMs (2, 3). Single-fraction SRS is favorable for small to medium sized AVMs in deep or eloquent locations, given the potential for high neurologic morbidity with surgery or embolization (4, 5).

The majority of literature related SRS treatment of AVMs is in the Gamma Knife setting. Data on frameless robotic radiosurgery (CyberKnife) treatment of AVMs is sparse, though results are comparable to the Gamma Knife system (6, 7). Less information is available on the appropriate radiation dosage for AVMs using the CyberKnife frameless radiosurgery system. Single-fraction dosages between 16 and 25 Gy have been recommended for the treatment of intracranial AVMs (6, 7). Often, radiosurgeons must balance the risks of undertreating an AVM versus overdosing organs at risk or eloquent brain parenchyma, leading to adverse radiation effects and the potential for permanent neurologic injury. Our retrospective study represents a single-center pilot study of patients with both ruptured and unruptured intracranial AVMs who underwent treatment with the CyberKnife using non-invasive CTA and MRI imaging.

## Methods

### Ethics

The studies involving human participants were reviewed and approved by the Institutional Review Board (IRB) at Medstar Georgetown University Hospital under the IRB code 2011-558. The patients/participants provided written informed consent to treatment.

### Patient Selection and Treatment

**Figure 1:**
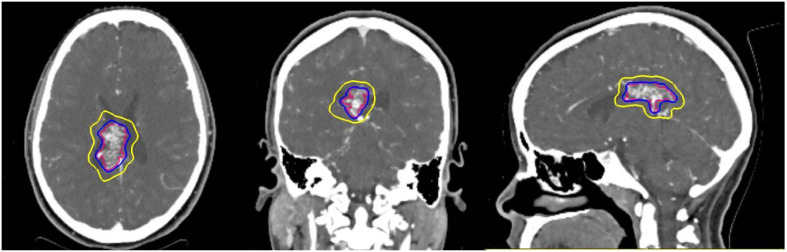
Representative stereotactic radiosurgery case with planned treatment volume (red) isodose (blue) and 50% isodose (yellow) can be seen in three planes on pretreatment planning CTA.

A retrospective review was performed of patients diagnosed with intracranial AVMs using diagnostic angiograms who were treated with CyberKnife SRS from July 20, 2006 to March 11, 2013 at Medstar Georgetown University Hospital. Patients were included if they were treated with single fraction SRS with or without pre-operative embolization. All patients in the study were evaluated by a multidisciplinary team of board-certified radiation oncologists, neuroradiologists, and neurosurgeons. CTA with MRI images were obtained for patients and used in pre-treatment planning for all AVM target volumes and critical structures were manually contoured by the radiation oncologist and neurosurgeon conjointly (**Figure 1**). Contours were routinely reviewed by neuroradiology (FB) prior to proceeding with treatment planning. Dose was determined at the discretion of a single radiation oncologist (BC) in consultation with the treating neurosurgeon and was dictated by the AVM nidus volume, intracranial location (eloquent versus non-eloquent) and proximity to critical normal structures. Normal tissue dose constraints were utilized per Benedict et al. (8). Treatment plans were generated using the CyberKnife treatment software (Multiplan, Accuray, Sunnyvale, CA, USA) inverse planning method.

### Outcomes Assessment

Follow up was performed by the interdisciplinary treating team per routine institutional practice. Serial surveillance CTA, MRI, and catheter-associated angiography were used to confirm AVM obliteration and progression unless acute changes in neurological status or symptoms warranted more immediate evaluation. Complete closure was defined as total obliteration of the AVM nidus and draining veins on surveillance radiological imaging. Partial closure was defined as a decrease in size of the AVM nidus despite persistence of the draining veins.

### Statistical Analysis

Statistical analysis was performed in order to identify pre-treatment and peri-treatment variables correlating with AVM obliteration following CyberKnife treatment. Descriptive characteristics tables were constructed using Student’s t-test for continuous variables and Chi-squared or Fischer exact tests for categorical variables. A univariate analysis of patient, AVM, and treatment characteristics was performed and stratified based on AVM response rate using the Kruskal-Wallis test, a non-parametric equivalent of the ANOVA. For each analysis, alpha was set to 0.05 with confidence intervals of 95% representing statistical significance. Median values and interquartile ranges (IQR), which is a more robust measure of dispersion, were reported for each variable. All statistical analyses were performed in R statistical software (version 3.4.2; R Foundation for Statistical Computing, Vienna, Austria).

## Results

### Patient and Treatment Characteristics

A total of 20 patients were retrospectively identified to have undergone SRS for intracranial AVMs with baseline characteristics described in **Table 1**. In our cohort, 13 (65%) patients were male, with a median age at treatment of 45 years (IQR, 26.75–51.0). Ten patients (50%) had prior history of hemorrhage and seven (35%) of the patients had pre-radiation interventions that included six (85.7%) embolization and one (14.3%) emergent decompressive hemicraniectomy with clip placement of the feeding vessels. The Spetzler-Martin grades of the AVMs treated included five Grade I (25%), seven Grade II (35%), seven Grade III (35%), and one Grade IV (5%) with a median nidus diameter of 1.8 cm (IQR, 1.05–2.55 cm) and a median AVM nidal volume of 4.38 cc (IQR, 1.8–8.69 cc).

**Table 1:** Descriptive Characteristics of CyberKnife Treated AVMs.

Parameters	Total (n = 20)
**Age at Treatment** (median, 25%-75% IQR)	*45 (26.75–51.0)*
**Sex** (n, %)	*—*
Male	*13 (65.0)*
Female	*7 (35.0)*
**Nidus Max Diameter** (median, 25%-75% IQR)	*1.80 (1.05–2.55)*
**AVM Volume** (median, 25%-75% IQR)	*4.38 (1.8–8.69)*
**Spetzler-Martin Grade** (n, %)	
1	*5 (25.0)*
2	*7 (35.0)*
3	*7 (35.0)*
4	*1 (5.0)*
**Prior Hemorrhage** (mean, %)	*10 (50.0)*
**Pre-Treatment Interventions** (n, %)	*—*
Embolization	*6 (30.0)*
Surgery	*1 (5.0)*
**Pre-Treatment Seizures** (n, %)	*11 (55.0)*
**Pre-Treatment Headaches** (n, %)	*11 (55.0)*
**Prior/Current Smokers** (n, %)	*4 (20.0)*
**Median Radiation Dose** (median, 25%-75% IQR)	*1800 (1750–1850)*
**Median Isodose Line** (median, 25%-75% IQR)	*0.83 (0.80–0.85)*
**Post-Treatment Hemorrhage** (n, %)	*1 (5.0)*
**Post-Treatment Seizure** (n, %)	*3 (15.0)*
**Complete Obliteration of AVM** (n, %)	*—*
Yes	*13 (65.0)*
Partial	*3 (15.0)*
Unknown	*4 (20.0)*
**Toxicity** (n, %)	*—*
None	*18 (90.0)*
Yes	*2 (10.0)*
**Simulation Time, weeks** (median, 25%-75% IQR)	*5.7 (3.0–9.9)*
**Follow-up Time, months** (median, 25%-75% IQR)	*42 (38.0–50.0)*

Stereotactic radiosurgery treatment planning consisted of a simulation prior to radiosurgery with a median time of 5.7 weeks (IQR, 3.0–9.9 weeks) between simulation and completion of SRS. These patients were treated with a median dose of 1800 cGy (range, 1,600–2,200 cGy) and a median prescription isodose line of 83% (IQR, 79–85%). Median follow-up time was 42 months (IQR, 38.0–50.0 months). Four patients (20%) were excluded from further analysis for the following reasons: two were lost to follow-up (10%), one patient was deceased by four years due to other causes (5%), and one patient underwent surgery (5%) prior to the four-year follow-up. The patient who underwent surgery received an angiogram at 28 months that showed a decrease in the size of the AVM nidus in addition to decreased shunting. The patient, however, did not wish to further observe the AVM and elected for immediate surgical resection.

### AVM Closure Rates

**Figure 2:**
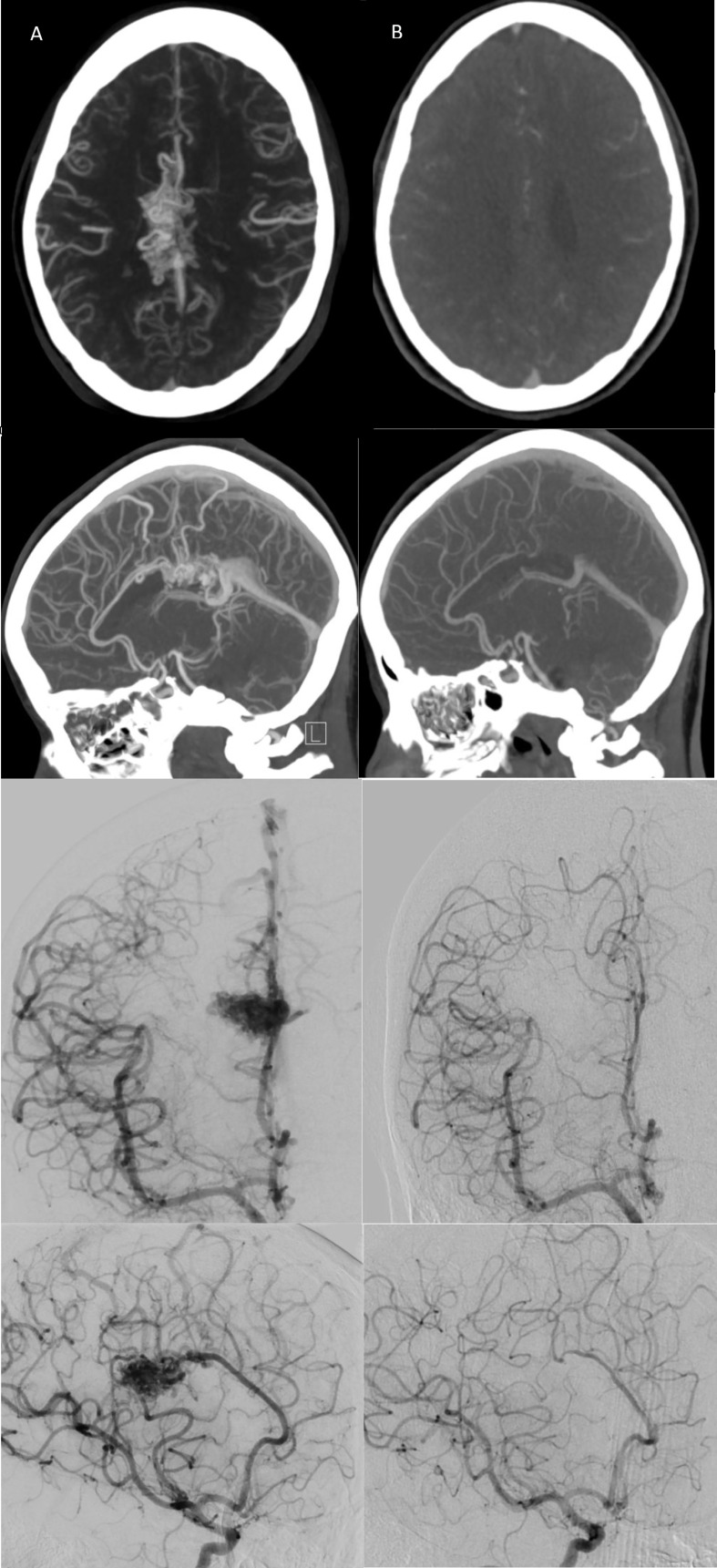
**(A)** Pre-radiosurgery, and **(B)** post-radiosurgery axial/sagittal CT angiogram and coronal/sagittal right ICA injection conventional angiograms for a representative case with follow-up images taken at 3 years post-CyberKnife treatment demonstrating complete AVM nidal obliteration with no residual draining vein(s).

At a median follow-up of 41.5 months (IQR, 37.25–50.5 months), there were a total of 16 patients with radiographically confirmed AVM obliteration or hemorrhage (**Figure 2**). Thirteen patients had complete obliteration of their AVM (81.3%) and three patients had partial closure (18.8%) (**Table 2**). Of the patients receiving 1800 cGy, all demonstrated complete obliteration. Partial obliteration occurred in two out of the five (40.0%) patients who received a dose of ≤ 1700 cGy. The decision to decrease the radiation dose in these cases was owed to the proximity of the AVM nidus to an eloquent area of the brain. Median time to radiographically confirmed AVM closure was 24 months (range, 22–36 months). Larger nidus max diameter (p = 0.0219) and higher Spetzler-Martin grade (p = 0.02833) demonstrated a statistically significant difference between partial and complete obliteration rates. However, age (p = 0.1386), nidal volume (p = 0.1939), prescription isodose line (p = 0.6345), radiation dose (p = 0.359), pre-SRS intervention (p = 0.1495), planning time between simulation and radiosurgery (p = 0.4996), and follow-up time (p = 0.8928) did not influence closure rates of AVMs (**Table 3**).

**Table 2:** Descriptive Characteristics of AVM Response Rate.

Parameters	Total (n = 16)	Partial Response (n = 3)	Complete Response (n = 13)	p-value
**Age at Treatment** (median, 25%-75% IQR)	*39 (25.75–52.5)*	*63 (45–67.5)*	*37 (25–49)*	*0.144*
**Sex** (n, %)	*—*	*—*	*—*	*0.545*
Male	*11 (61.5)*	*3 (100.0)*	*8 (61.5)*	*—*
Female	*5 (38.5)*	*0 (0.0)*	*5 (38.5)*	*—*
**Nidus Max Diameter** (median, 25%-75% IQR)	*1.4 (1.0–2.5)*	*3.3 (2.85–3.45)*	*1.1 (1–1.8)*	0.008*
**AVM Volume** (median, 25%-75% IQR)	*4.38 (0.96–9.89)*	*9.14 (6.58–12.69)*	*3.5 (0.91–8.84)*	*0.128*
**Spetzler-Martin Grade** (median, 25%-75% IQR)	*—*	*—*	*—*	*0.064*
1	*4 (25.0)*	*0 (0.0)*	*4 (30.8)*	*—*
2	*5 (31.2)*	*0 (0.0)*	*5 (38.5)*	*—*
3	*6 (37.5)*	*2 (66.7)*	*4 (30.8)*	*—*
4	*1 (6.2)*	*1 (33.3)*	*0 (0.0)*	*—*
**Prior Hemorrhage** (mean, %)	*10 (62.5)*	*1 (33.3)*	*9 (69.2)*	*0.620*
**Pre-Treatment Interventions** (n, %)	*6 (37.5)*	*0 (0.0)*	*6 (46.2)*	*0.408*
**Pre-Treatment Seizures** (n, %)	*2 (12.5)*	*0 (0.0)*	*2 (15.4)*	*1.000*
**Pre-Treatment Headaches** (n, %)	*7 (43.8)*	*1 (33.3)*	*6 (46.2)*	*1.000*
**Median Radiation Dose** (median, 25%-75% IQR)	*1800 (1700–1850)*	*1700 (1650–1850)*	*1800 (1800–1800)*	*0.527*
**Median Isodose** (median, 25%-75% IQR)	*0.83 (0.79–0.85)*	*0.81 (0.78–0.83)*	*0.83 (0.8–0.85)*	*0.824*
**Post-Treatment Hemorrhage** (n, %)	*1 (6.2)*	*1 (33.3)*	*0 (0.0)*	*—*
**Post-Treatment Seizure** (n, %)	*3 (18.8)*	*1 (33.3)*	*2 (15.4)*	*0.408*
**Toxicity** (n, %)	*—*	*—*	*—*	*1.000*
None	*13 (81.2)*	*2 (66.7)*	*11 (84.6)*	*—*
Yes	*3 (18.8)*	*1 (33.3)*	*2 (15.4)*	*—*
**Simulation Time** (median, 25%-75% IQR)	*5.5 (2.75–10.0)*	*3 (2–7)*	*6 (4–10)*	*0.530*
**Follow-up Time** (median, 25%-75% IQR)	*41.5 (37.25–50.5)*	*49 (42–49.5)*	*40 (38–52)*	*0.753*

* Significant Values (p < 0.05).

**Table 3:** Kruskall-Wallis Rank Test of AVM Response Rate.

Parameters	Partial Response (n=3)	Complete Response (n=13)	p-value
**Age, mean (sd)**	*54.00 (23.81)*	*36.85 (15.98)*	*0.1386*
**Nidus Max Diameter, mean (sd) **	*3.10 (0.62)*	*1.48 (0.86)*	0.0219*
**Nidal Volume, mean (sd)**	*9.80 (6.13)*	*4.88 (4.38)*	*0.1939*
**Spetzler-Martin Grade, n (%)**	*—*	*—*	0.02833*
1	*0 (0.0)*	*4 (30.8)*	*—*
2	*0 (0.0)*	*5 (38.5)*	*—*
3	*2 (66.7)*	*4 (30.8)*	*—*
4	*1 (33.3)*	*0 (0.0)*	*—*
**Dose, mean (sd)**	*1766.67 (208.17)*	*1838.46 (166.02)*	*0.359*
**Isodose, mean (sd)**	*0.80 (0.05)*	*0.81 (0.06) *	*0.6345*
**Intervention, n (%)**	*0 (0.0)*	*6 (46.2)*	*0.1495*
**Simulation Time, mean (sd)**	*5.00 (5.29)*	*7.31 (5.65)*	*0.4996*
**Follow-up, mean (sd)**	*44.67 (8.39)*	*49.15 (23.28)*	*0.8928*

* Significant Values (p < 0.05).

### Neurological Deficits and Toxicity

Post-treatment hemorrhage was seen in one patient (5.0% of the total population) with a relatively large (volume = 16.23 cc) frontoparietal AVM five months following CyberKnife treatment. This hemorrhage caused left hemiplegia. Surgical resection of the AVM was then performed, with preoperative embolization. A second patient experienced focal visual field loss 42 months following radiosurgery with 2200 cGy to a small left parietal-occipital AVM (volume = 0.55 cc). Angiography at the time of these brief vision changes demonstrated complete obliteration of the AVM nidus.

## Discussion

A number of studies have explored the use of Gamma Knife for the treatment of intracranial AVMs and more specifically the optimal dose required for complete obliteration. One such study conducted by Ding et al. was composed of 938 patients with unruptured AVMs who were treated with a mean radiosurgical dose of 21 Gy. In this study, the median nidus volume was 2.4 cm^3^ with 57% of the population having a Spetzler-Martin grade of 3 or greater. With a mean of follow-up time of 71 months (9), obliteration was achieved in 65% of patients. Of note, Ding et al. demonstrated a dose of ≥20 Gy for the Gamma Knife system yielded more robust obliteration rates compared to patients treated with doses <20 Gy (70% vs 36%; P <.001). Flickinger et al. reported the results of a series including 351 AVM patients treated with the Gamma Knife system and demonstrated an obliteration rate of 75% (10). The median marginal dose administered in the Flickinger study was 20 Gy (range, 12–30 Gy). Similar to Ding et al, Flickinger et al. found that doses greater than 20 Gy resulted in a statistically significant increase obliteration of treated AVMs (p <0.001). Another study of 755 patients with AVMs who underwent single-fraction treatment with Gamma Knife, demonstrated that 55 patients (6%) in their cohort developed radiation-related toxicity at a median follow-up time of 75 months (11). Thirty-six of these patients developed transient neurological events that included hemiparesis, headaches, seizure(s), sensory dysfunction, cerebellar ataxia, memory-loss, and facial and ocular movement disorder. Nineteen patients developed permanent symptoms such as hemiparesis, reduced consciousness, visual, and ocular motor deficits. The median target volume in this study was larger than the aforementioned trials at 3.6 cm^3^ (0.1–26.3 cm^3^) with a median margin dose was 20 Gy (13–27 Gy). The adverse event rates were found to be 3.2%, 5.8%, 6.7%, and 7.5% at 1, 2, 3, and 5 years, respectively. Factors that predicted an increase in adverse radiation events included higher margin radiation dose, larger AVM volume, and higher Spetzler–Martin grade, which were all factors found in the present study to be nominally but not significantly associated with increased risk of toxicity.

Data regarding use of CyberKnife in the treatment of cerebral AVMs has thus far been very limited with only a single study exploring long-term outcomes in the literature (2, 12–15). This analysis by Colombo et al. described a cohort of 279 patients diagnosed with intracranial AVMs treated with CyberKnife (2). Within this cohort, 102 patients had follow-up of more than 36 months and were included in the analysis. In those patients with longer term follow up, 80 underwent angiographic evaluation with 65 patients (81.3%) demonstrating complete obliteration of the AVM nidus. The AVM volumes ranged from 0.1 – 42 ml in size with minimum radiation doses of 15 to 23 Gy (median dose of 18 Gy). Post-operative complications included one case of transient difficulties with speech and acalculia that resolved with a short-term corticosteroid, and in addition eight patients developed hemorrhagic events leading to one death and one case of quadriparesis. This study demonstrated a strong inverse relationship between complete obliteration and both AVM nidal volume and Spetzler-Martin grade.

Our team began treating AVMs with the CyberKnife system in 2002. A non-invasive technique using IV bloused CTA/MRI for treatment planning and simulation was developed and implemented at our institution, which has been previously described (12). Traditionally, angiography has been the mainstay in both the diagnosis and treatment of AVMs as it offers anatomically accurate visualization of the vasculature as well as options for immediate treatment (16–18). However, the use of angiography still involves invasive components that can lead to complications such as hematoma formation, vascular dissection, vasospasm or AVM rupture (16, 19). Several studies have demonstrated that CTA and MRA can be viable options for patients and can lead to equivalent diagnostic results compared to angiograms (20–23). Two separate studies by Willems et al. and Essig et al. have demonstrated that 4D-CTA can better delineate the AVM nidus when compared to traditional angiography (24, 25). Additional studies have also demonstrated improved temporal resolution using 4D-CTA compared to angiography which can help distinguish the collateral and feeding vessels of the AVM (26–28). The benefits of the both these features in the radiosurgical planning is that it allows the physician to limit the treatment to the AVM nidus and spare the surrounding cerebral parenchyma decreasing the effects of radiation toxicity. While larger studies are needed to further elucidate and develop specific protocols for work-up and treatment of AVMs using CTA/MRA, this study supports the position that non-invasive procedures can be acceptable and safe alternatives.

We now report the mature results of our pilot analysis. In the present study, frameless SRS with CyberKnife using non-invasive CTA and MRI imaging appears to be both a safe and effective technique for the treatment of intracranial AVMs. Additionally, our data and experience suggest that a single dose of 1800 cGy offers a high-rate of nidus obliteration in small to medium sized AVMs, while avoiding the deleterious neurologic toxicity often seen with higher doses. One patient who developed complications following SRS was treated at a higher dose level of 2200 cGy. However, this risk of treatment-related toxicity must be balanced with a high enough radiation dose to yield adequate nidus obliteration. In our experience, doses ≤ 1700 cGy provide an inadequate response and resulted in only partial closure of the AVM nidus in two out of five patients (40.0%) at four years. Therefore, it is imperative that a sufficient radiation dose be given in order to effectively ablate these AVMs while limiting the radiation exposure to the surrounding normal cerebral parenchyma. Our results show that 81.3% of our evaluable patients demonstrated complete obliteration within the four-year time frame we established for follow-up. Of the patients receiving 1800 cGy, 100% were found to have a complete closure of the AVM nidus within four-years and no patients demonstrated adverse effects of the radiotherapy at the time of analysis. The high level of closure in our present study is likely the result of our relatively lower Spetzler-Martin grading (56.3% of our patients have a grade of ≤ 2), small nidal volumes (median nidal volume of 4.38 cc), and AVM diameters (median nidal diameter of 1.8 cm).

Finally, it is possible that our satisfactory clinical outcomes could be attributed to our comparatively extended treatment planning times (median 5.5 weeks) giving our team more than adequate time to precisely delineate the target volume while diligently identifying critical structures to minimize toxicity. It is routine for the angiogram, treatment planning, and radiosurgery to occur within the same day when using framed SRS platforms. A potential advantage of frameless SRS is that the treatment planning, quality assurance, and delivery can be performed over a longer period of time. At present, there are no studies that look at treatment planning time and their effects on AVM closure rates as many centers opt to simulate, plan, and treat patients on the same day. However, our analysis suggests that in the case of intracranial AVMs, treatment can be delayed for weeks after simulation without adversely effecting outcomes.

## Study Limitations

This study has limitations, which have implications for its interpretation and generalizability. The small number of patients in our cohort and the fact that it is based out of a single institution limits the generalizability to other centers and patient populations not represented in our study. Furthermore, 20% of our population were not evaluable at 4 years. Therefore, our complete obliteration rate at 4 years may have been as low as 65% rather than our reported 81.3%. These numbers demonstrate the importance of increasing our study population and retaining patients for follow-up purposes whenever possible. However, given the pilot nature of the data, this is to be expected with future larger potentially multi-institution studies addressing these critiques.

## Conclusion

This mature pilot analysis of 20 patients with intracranial AVMs treated with CyberKnife using non-invasive CTA and MRI imaging demonstrates the efficacy and safety of this procedure in the treatment of cerebral AVMs, and provides insight into the challenges faced with radiosurgical obliteration of this disease entity. Our study suggests that acceptable obliteration rates can be achieved without compromising patient safety using only CTA and MRI imaging, longer treatment planning times, and lower doses of radiation relative to more traditional methods. At present, there is little data supporting specific dosing with CyberKnife in the treatment of intracranial AVMs and the relation that this dosing has with post-radiation toxicity. We demonstrate that adequate obliteration can be achieved with 1800 cGy using CyberKnife while also limiting the radiation dose to the surrounding cerebral parenchyma and minimizing adverse side effects. This study also suggests that spending additional time planning treatments now possible with frameless systems may improve obliteration rates and decrease radiation toxicity. With the growing use of radiosurgery in the treatment of these and other intracranial diseases, it is important to continue to further our understanding of the techniques we use in the hopes of providing more robust and enhanced care to our patients. In the future, additional large cohort studies are needed to better characterize and confirm our findings and validate this approach in the treatment of intracranial AVMs.

## Data Availability Statement

The datasets presented in this article are not readily available in order to protect patient privacy. Requests to access the datasets should be directed to Ryan Kelly, Rmk78@georgetown.edu.

## Ethics Statement

The studies involving human participants were reviewed and approved by the Institutional Review Board (IRB) at Medstar Georgetown University Hospital under the IRB code 2011-558.

## Author Contributions

BC was responsible for formulation of the project as well as data collection, manuscript editing, and final revisions. RK and AC were responsible for data collection, data analysis, and manuscript writing and editing. MN, J-MV, AA, SC, CK, AS, JM, RA, JL, FB, VN, and KM contributed to manuscript editing and final revisions. All authors contributed to the article and approved the submitted version.

## Conflict of Interest

BC, JL and SC have received research funding from Accuray for previous work and current ongoing research not related to this topic.

The remaining authors declare that the research was conducted in the absence of any commercial or financial relationships that could be construed as a potential conflict of interest.
